# Microbial etiology and antimicrobial resistance patterns in neonates admitted to a tertiary neonatal intensive care unit in Mogadishu, Somalia

**DOI:** 10.3389/fped.2026.1888678

**Published:** 2026-08-18

**Authors:** Ali Kutta ÇELİK, Tigad Abdisad Ali, Farah Abdullahi Ismail, Suad Abdikarim ISSE, Mucahit TÜRKAN, Abdikarim Abdi Adam, Liban Ade Hussein, Edanur Yeşil, Mohammed A. M. Ahmed

**Affiliations:** 1 Department of Infectious Diseases and Clinical Microbiology, Mogadishu-Somalia-Turkiye Recep Tayyip Erdoğan Training and Research Hospital, Mogadishu, Somalia; 2 Department of Infection Prevention Control (IPC), Turkey Recep Tayyip Erdoğan Training and Research Hospital, Mogadishu, Somalia; 3 Tropical Medicine and Infectious Diseases, School of Postgraduate Studies, Benadir University, Mogadishu, Somalia; 4 Department of Pediatrics, Mogadishu-Somalia-Turkiye Recep Tayyip Erdoğan Training and Research Hospital, Mogadishu, Somalia; 5 Department of Environmental Health and Climate, IPC Section, Ministry of Health and Human Services, Mogadishu, Somalia; 6 Department of Medicine, Faculty of Medicine and Surgery, Salaam University, Mogadishu, Somalia; 7 Department of Pediatric Infectious Diseases, Mersin University Faculty of Medicine, Mersin, Türkiye; 8 Department of Paediatrics, Faculty of Medicine and Surgery, Mogadishu University, Mogadishu, Somalia; 9 Department of Paediatric & Congenital Cardiology, Mogadishu Heart Center, Mogadishu, Somalia

**Keywords:** antimicrobial resistance, *Candida* spp, *Gram-negative bacteria*, *Gram-positive bacteria*, multidrug resistance, neonatal intensive care unit, neonatal sepsis

## Abstract

**Objective:**

Neonatal sepsis continues to be one of the leading causes of morbidity and death, particularly in low- and middle-income countries (LMICs). This study aimed to evaluate the microbiological etiology, temporal trends, and antibiotic resistance patterns in a tertiary neonatal intensive care unit (NICU) over a six-year period.

**Methods:**

This retrospective study included neonates admitted to a tertiary neonatal intensive care unit between 2018 and 2024. Early-onset infections were defined as infections occurring within the first 72 hours of life, while late-onset infections were defined as infections occurring after 72 hours. Logistic regression analysis was used to assess temporal trends and identify factors associated with multidrug-resistant infection.

**Results:**

Microbial growth was observed in 891 (30.0%) of the total samples. A total of 891 isolates (30.0% of all samples) with complete data were included in the final study after duplicate and contaminated isolates were removed. Among these isolates, 472 (53.0%) were *Gram-positive bacteria*, 366 (41.1%) were *Gram-negative bacteria*, and 53 (5.9%) were *Candida spp. isolates*. isolates. In addition to a greater proportion of *Gram-negative organisms* (43.6% vs. 35.4%) and *Candida species* (7.5% vs. 2.6%), late-onset infections showed considerably higher culture-positive rates (37.7% vs. 20.7%, *p* < 0.001) than early-onset infections. On the other hand, *Gram-positive* organisms predominated in early-onset infections (62.0% vs. 48.9%, *p* < 0.001). In Multivariate logistic regression analysis, study year was not independently associated with MDR after adjustment (aOR: 1.06, 95% CI: 0.98–1.14, *p* = 0.169), whereas late-onset infection was still independently linked with MDR infection (aOR: 2.53, 95% CI: 1.84–3.47, *p* < 0.001). Despite changes in MDR rates throughout the course of the research years, *carbapenem resistance* demonstrated a significant growing trend over time (*p* for trend = 0.004). Notably, none of the commonly used empirical antibiotics met the recommended susceptibility level of ≥80%.

**Conclusion:**

This six-year study reveals a significant burden of neonatal infections in the NICU, with *Candida spp.* and *Gram-negative* organisms predominating in late-onset infections and Gram-positive organisms in early-onset infections. *Carbapenem resistance* dramatically increased over time, and late-onset infection was independently linked to multidrug resistance. These results highlight the necessity of stronger IPC, localized antimicrobial stewardship, updated empirical treatment guidelines, and regular AMR surveillance.

## Introduction

Neonatal infections remain a major contributor to morbidity and mortality worldwide, with the greatest burden occurring in low- and middle-income countries (LMICs), where access to timely diagnosis, effective antimicrobial therapy, neonatal intensive care, and infection prevention services is often limited (1, 2). Bacterial infections, including sepsis, pneumonia, and meningitis, are responsible for an estimated 550,000 neonatal deaths annually (1). Although global neonatal mortality has declined, progress remains uneven. In sub-Saharan Africa, neonatal mortality is approximately 27 per 1,000 live births, while estimates for Somalia remain substantially higher at 34–36 per 1,000 live births, far above the Sustainable Development Goal target of fewer than 12 per 1,000 live births (11, 12).

The clinical diagnosis of neonatal sepsis is challenging because presenting features are often nonspecific and currently available biomarkers have limited predictive accuracy (13). In Somalia, these challenges are further intensified by structural constraints, including limited access to healthcare, inadequate neonatal intensive care unit (NICU) infrastructure, shortages of trained personnel, and weak infection prevention and control (IPC) capacity (12, 14). Recent national evidence from the 2024 Harmonized Health Facility Assessment showed that only 24.5% of healthcare facilities had standard precaution guidelines, 18.5% had healthcare waste management guidelines, and 21.4% had staff trained in waste management [20]. Similarly, a nationwide healthcare waste management assessment reported major gaps in waste segregation and unsafe disposal practices, including open burning and unprotected pit burning of infectious waste (21). Such system-level weaknesses may increase the risk of healthcare-associated infections and compromise neonatal patient safety, particularly in fragile health-system settings (51).

Neonatal infections are commonly classified as early-onset infections, occurring within the first 72 hours of life, and late-onset infections, occurring thereafter (3). This distinction is clinically important because the two syndromes often differ in acquisition pathways, risk factors, pathogen distribution, and antimicrobial resistance profiles (3, 4). Early-onset infections are commonly associated with maternal and perinatal factors, including maternal colonization, prolonged rupture of membranes, prematurity, intrapartum fever, and exposure during delivery (3). By contrast, late-onset infections are more frequently linked to postnatal and healthcare-associated exposures, including prolonged hospitalization, invasive procedures, vascular access devices, mechanical ventilation, broad-spectrum antibiotic exposure, overcrowding, and deficiencies in IPC practices (4, 5).

Antimicrobial resistance (AMR) is an increasing threat to neonatal infection management, particularly in NICUs, where critically ill neonates are frequently exposed to broad-spectrum antibiotics and prolonged inpatient care (5, 6). Resistance to commonly used empirical agents, including ampicillin, gentamicin, and third-generation cephalosporins, has been widely reported in LMIC settings, raising concerns about the continued effectiveness of standard neonatal sepsis regimens (5, 6, 7, 15, 16). Gram-negative organisms are especially concerning because of resistance mechanisms such as extended-spectrum beta-lactamase production and carbapenem resistance, which substantially narrow therapeutic options for severe neonatal infections (5, 8, 22–24). The World Health Organization has emphasized the growing global burden of antibiotic resistance and the need for strengthened microbiological surveillance, local antibiograms, antimicrobial stewardship, and integration of AMR monitoring with IPC programs (6–8, 17–19).

Despite the expansion of hospital-based neonatal and critical care services in Mogadishu, neonatal-specific microbiological and AMR data from Somalia remain scarce (8, 9). Existing Somalia-focused evidence has highlighted gaps in routine microbiology, unit-level antibiograms, IPC implementation, and antimicrobial stewardship, particularly in intensive care settings (9, 10). However, the limited availability of NICU-based data constrains the identification of dominant pathogens, monitoring of resistance trends, and development of locally appropriate empirical treatment recommendations. Therefore, this study aimed to assess the microbiological etiology, temporal trends, and antibiotic resistance patterns among neonates admitted to a tertiary NICU in Mogadishu, Somalia, over a six-year period. By characterizing pathogen distribution according to infection onset and evaluating resistance patterns over time, this study provides locally relevant evidence to inform empirical antibiotic selection, AMR surveillance, IPC strengthening, and neonatal antimicrobial stewardship interventions.

## Materials and methods

### Study design and setting

This retrospective observational study was conducted in the neonatal intensive care unit (NICU) of the Mogadishu Somali-Turkey Recep Tayyip Erdoğan Training and Research Hospital. Medical records of neonates admitted between January 2018 and December 2024 were reviewed.

### Patient and sample selection

Microbiological cultures were performed only in patients with clinical suspicion of sepsis; routine culture sampling was not conducted in asymptomatic patients. Clinical specimens included blood cultures, urine cultures, and deep tracheal aspirate samples. Specimen collection was guided by the patient's clinical presentation and the judgment of the treating physician. Neonates were considered to have suspected sepsis when clinical manifestations and/or laboratory abnormalities warranted microbiological investigation by the attending pediatrician. Clinical evaluation incorporated signs suggestive of systemic infection together with supportive laboratory findings, including leukocytosis, leukopenia, thrombocytopenia, and elevated inflammatory markers. Therefore, the study population represents a selected high-risk group rather than the entire NICU population.

### Inclusion and exclusion criteria

All neonates with suspected sepsis from whom culture samples were obtained were included in the study. To avoid over-representation of the same infectious episode, repeated isolates obtained from the same patient during a single infection episode were excluded. When multiple cultures yielded the same organism within the same episode, only the first isolate was retained. Contaminant cultures and records with incomplete microbiological or antimicrobial susceptibility data were also excluded. The final analytical cohort, therefore, consisted of unique clinically relevant isolates.

Contamination was defined as the isolation of common skin flora (e.g., *coagulase-negative staphylococci*) in a single culture without accompanying clinical or laboratory evidence of infection.

### Laboratory methods

Blood culture samples were processed using the AutobioR BC60 blood culture system. Due to the absence of automated identification systems, microorganisms detected in culture-positive media were identified manually using conventional microbiological and biochemical methods.

Antimicrobial susceptibility testing was performed using disk diffusion and E-test methods, with standard commercial antibiotic discs, and results were interpreted according to the Clinical and Laboratory Standards Institute (CLSI) criteria. Only isolates with available antimicrobial susceptibility data (excluding *Candida spp*.) were included in the resistance analysis. The antibiotic discs used for susceptibility testing included *imipenem* (10 μg), *meropenem* (10 μg), *colistin* (10 μg), *piperacillin–tazobactam* (100/10 μg), *ampicillin–sulbactam* (10/10 μg), *ceftriaxone* (30 μg), *gentamicin* (10 μg), *vancomycin* (30 μg), *teicoplanin* (30 μg), and *clindamycin* (2 μg), together with other antimicrobial agents routinely used in the laboratory according to the microorganism identified.

Cases of candiduria without accompanying pyuria and *Candida* spp. isolated from deep tracheal aspirate samples were not considered true pathogens and were excluded to minimize misclassification of colonization as infection in non-sterile sites. Species-level identification and antifungal susceptibility testing for *Candida* spp. were not available; therefore, all isolates were reported as *Candida* spp.

### Definitions

Sepsis was defined as a systemic infection supported by clinical findings in conjunction with laboratory indicators and/or microbiological evidence obtained from sterile body fluids. Culture-positive cases were classified as **confirmed sepsis**, whereas cases with compatible clinical and laboratory findings but negative cultures were considered **probable sepsis**.

Based on the timing of onset, infections (6, 10, 26–30) were categorized as:

- **Early-onset sepsis (EOS):** occurring within the first 72 h of life
- **Late-onset sepsis (LOS):** occurring after 72 h

MDRwas defined according to the criteria proposed by Magiorakos et al. (31), whereby isolates exhibiting resistance to at least one agent in three or more antimicrobial classes were classified as MDR. To further characterize MDR patterns, antimicrobial susceptibility profiles of MDR-positive isolates were evaluated descriptively. For the predominant pathogens, the most frequently observed resistance patterns were summarized according to individual antimicrobial agents and are presented in Supplementary Table S2.

In neonates with suspected infection or clinical signs of sepsis, a stepwise empirical antibiotic treatment protocol was implemented, consistent with practices reported in similar settings (21, 22). The empirical antibiotic regimen in our NICU was structured as follows:

- **First-line therapy:** ampicillin plus gentamicin
- **Second-line therapy:** third-generation cephalosporins, piperacillin–tazobactam, and amikacin
- **Third-line therapy:** glycopeptides, linezolid, daptomycin, and ciprofloxacin

Fluconazole was administered in selected high-risk neonates, particularly those with persistent thrombocytopenia or prematurity. The choice of empirical therapy was individualized based on the patient's clinical condition, risk factors, and local antibiogram data.

### Statistical analysis

Statistical analyses were performed using SPSS version 27.0 (IBM Corp., Armonk, NY, USA).

Categorical variables were expressed as numbers (*n*) and percentages (%). The distribution of *Gram-positive* (GP), *Gram-negative* (GN), and *Candida* spp. Infections according to year, timing of infection onset, and sex were compared using the Pearson chi-square test or Fisher's exact test, as appropriate.Logistic regression analysis was used to evaluate the association between pathogen type and timing of infection onset, with results presented as odds ratios (ORs) and 95% confidence intervals (95% CIs) and illustrated using forest plots.

Multivariate logistic regression analysis was performed to identify independent factors associated with MDR. Variables included in the Multivariate model were selected based on clinical relevance and a univariate analysis threshold of *p* < 0.10. Adjusted ORs with 95% CIs were reported.

Temporal trends in MDR rates over the study period were analyzed using logistic regression, with year included as a continuous variable to assess linear trends; corresponding *p-values* for trend values were calculated.

All statistical tests were two-tailed, and a *p*-value <0.05 was considered statistically significant.

### Ethical approval

Ethical approval for this study was obtained from the Ethics Committee of the Mogadishu Somali-Turkey Training and Research Hospital (Approval No: MSTH:2174).

## Results

### Microbiological growth and isolate characteristics

A total of 2,966 clinical samples were taken from the NICU over the course of the six-year study period; 891 (30.0%) of these samples produced microbial growth and were used in the microbiological analysis. Of these isolates, 53 (5.9%) were isolates of *Candida spp*., 472 (53.0%) were *Gram-positive bacteria*, and 366 (41.1%) were *Gram-negative bacteria*. 536 (60.2%) and 355 (39.8%) of the culture-positive isolates came from male and female newborns, respectively.

### Early-onset and late-onset sepsis distribution

Based on the timing of infection onset, 274 isolates (30.8%) were classified as early-onset sepsis (EOS), while 617 isolates (69.2%) were classified as late-onset sepsis (LOS), as shown in Table 1.

**Table 1:** Baseline characteristics of the study population.

Variable	Category	*n* (%)
Total isolates		891
Sex	Female	355 (39.8)
	Male	536 (60.2)
Age at onset	EOS	274 (30.8)
	LOS	617 (69.2)

Values are presented as *n* (%).

EOS, early-onset sepsis; LOS, late-onset sepsis.

When EOS and LOS groups were compared, there was no statistically significant association between sex and timing of infection onset (*p* = 0.548). Blood culture was the most common specimen type in both EOS and LOS groups. However, urine samples were significantly more frequent among LOS cases compared with EOS cases (*p* < 0.001).

Culture-positive rates were also significantly higher among LOS cases than EOS cases, with rates of 37.7% and 20.7%, respectively (*p* < 0.001), as shown in Table 2.

**Table 2:** Comparison of clinical, microbiological, and culture-positivity characteristics between early-onset and late-onset neonatal sepsis episodes.

Variable	Category	≤72 h (*n* = 1,328)	>72 h (*n* = 1,638)	*p*-Value
Sex	Female (*n*, %)	511 (38.5%)	648 (39.6%)	0.548
Sample Type (*n*, %)	Blood	1,284 (96.7%)	1,504 (91.8%)	<0.001
	Deep tracheal aspirate	2 (0.2%)	8 (0.5%)	
	Urine	42 (3.2%)	126 (7.7%)	
Culture positivity	Growth present, *n* (%)	274 (20.7%)	617 (37.7%)	<0.001
Pathogen distribution^a^	Gram-negative (*n*, %)	97 (35.4%)	269 (43.6%)	<0.001
	Gram-positive (*n*, %)	170 (62.0%)	302 (48.9%)	
	*Candida spp*	7 (2.6%)	46 (7.5%)	

Values are presented as *n* (%). *P*-values were calculated using the Pearson chi-square test or Fisher's exact test where appropriate.

EOS, early-onset sepsis; LOS, late-onset sepsis.

a Pathogen distribution was analyzed among culture-positive isolates (EOS *n* = 274; LOS *n* = 617).

### Overall Pathogen Distribution

The overall distribution of pathogens. Depending on when an infection occurred, the distribution of germs changed dramatically. *Gram-positive* organisms were more common in EOS, while Candida spp. and *Gram-negative* organisms were more prevalent in LOS (Table 3).

**Table 3:** Distribution of infection types by year, age at onset, and sex.

Variable	Category	Gram-positive *n* (%)	Gram-negative *n* (%)	*Candida* spp *n* (%)	*p*-value
Year	2018	71 (57.3)	44 (35.5)	9 (7.3)	<0.001
	2019	111 (69.4)	48 (30.0)	1 (0.6)	
	2020	59 (62.8)	27 (28.7)	8 (8.5)	
	2021	100 (57.8)	71 (41.0)	2 (1.2)	
	2022	64 (43.8)	72 (49.3)	10 (6.8)	
	2023	9 (11.0)	60 (73.2)	13 (15.9)	
	2024	58 (51.8)	44 (39.3)	10 (8.9)	
Age at onset	EOS	170 (62.0)	97 (35.4)	7 (2.6)	<0.001
	LOS	302 (48.9)	269 (43.6)	46 (7.5)	
Sex	Female	187 (52.7)	147 (41.4)	21 (5.9)	0.987
	Male	285 (53.2)	219 (40.9)	32 (6.0)	

Values are presented as *n* (%). *P*-values were calculated using the Pearson chi-square test or Fisher's exact test where appropriate.

EOS, early-onset sepsis; LOS, late-onset sepsis.

### Association Between Infection Onset and Pathogen Type

The pathogen type and sex did not significantly correlate (*p* = 0.987). Over the course of the investigation, the distribution of pathogens varied significantly (*p* < 0.001). The majority of culture-positive isolates were identified as *coagulase-negative staphylococci* (*n* = 281), *Staphylococcus aureus* (*n* = 158), *Escherichia coli* (*n* = 154), and Klebsiella species (*n* = 114) (Table 8). LOS was linked to a higher incidence of both Gram-negative infection (odds ratio [OR]: 1.56; 95% CI: 1.16–2.10; *p* = 0.003) and Candida spp. infection (OR: 3.70; 95% CI: 1.63–8.37; *p* = 0.002) in univariate logistic regression analysis. On the other hand, LOS cases had a lower risk of Gram-positive infections (OR: 0.59; 95% CI: 0.44–0.78; *p* < 0.001).

### Predictors of Multidrug Resistance

**Figure 1:**
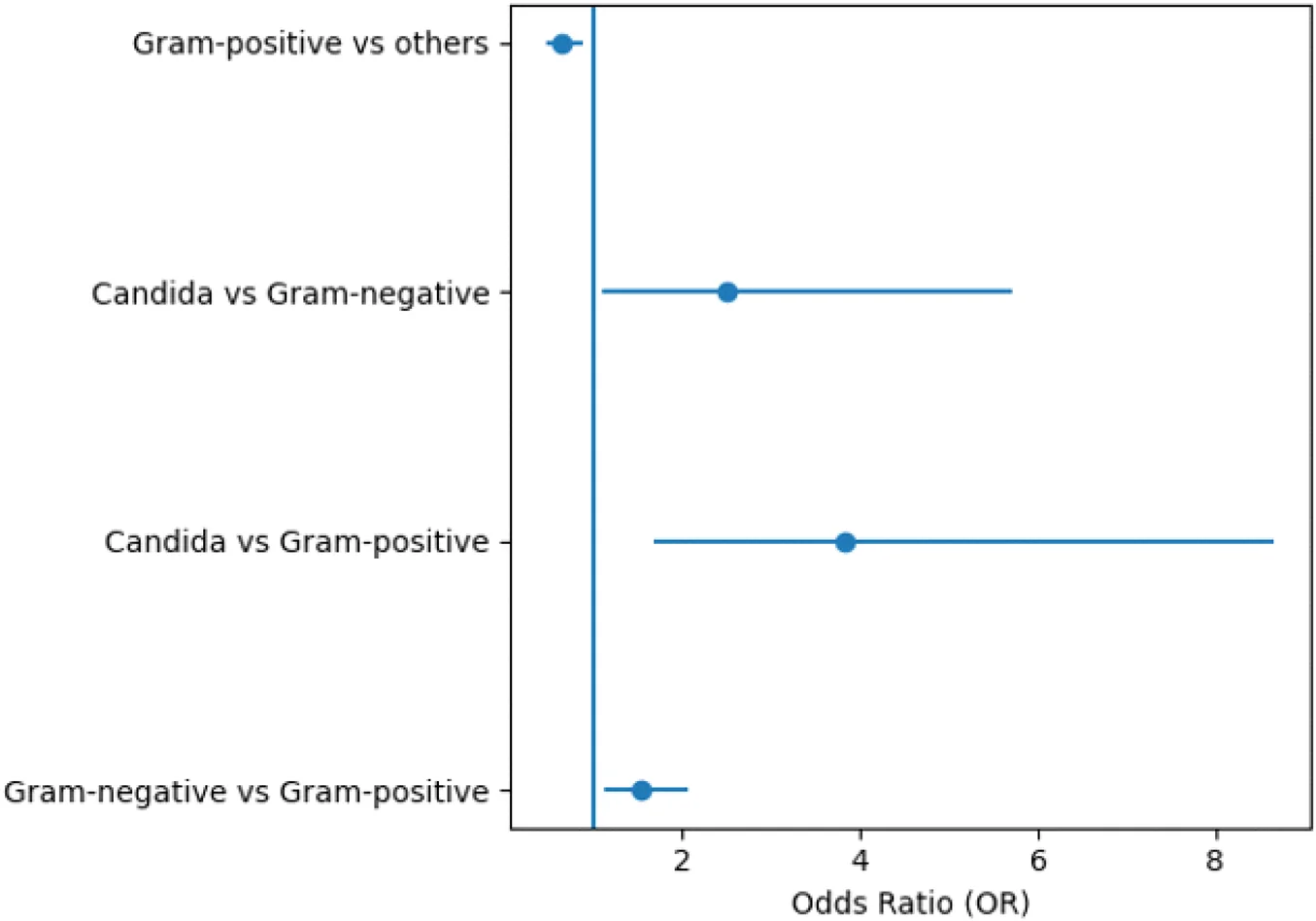
Forest plot showing the association between age at onset [early-onset sepsis [EOS] vs. late-onset sepsis [LOS]] and infection type. Forest plot showing the association between age at onset and infection type. Odds ratios (OR) and 95% confidence intervals (CI) were calculated using univariate logistic regression analysis. Results are presented relative to Gram-positive infections as the reference category. (EOS, early-onset sepsis, LOS, late-onset sepsis).

**Figure 2:**
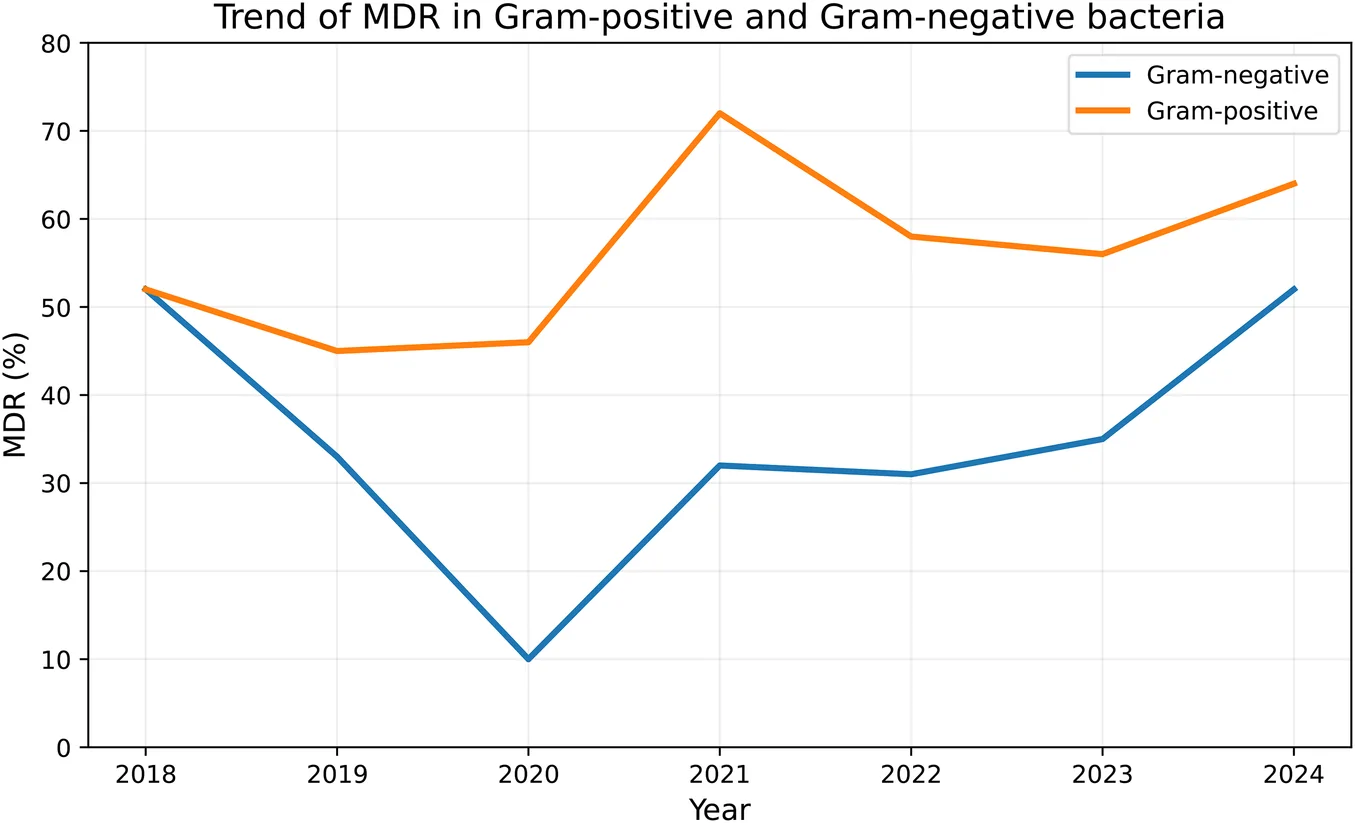
Temporal trends in multidrug resistance (MDR) rates among gram-positive and gram-negative organisms (2018–2024).

These results are shown in Figure 1 and Table 4. LOS was found to be an independent predictor of multidrug resistance (MDR) using Multivariate logistic regression analysis (aOR: 2.53; 95% CI: 1.84–3.47; *p* < 0.001). However, when controlling for relevant confounders, study year did not have an independent relationship with MDR (aOR: 1.06; 95% CI: 0.98–1.14; *p* = 0.169). There was no significant correlation between sex and MDR (aOR: 1.05; 95% CI: 0.79–1.41; *p* = 0.731), although MDR was less common in Gram-negative organisms than in Gram-positive organisms (aOR: 0.37; 95% CI: 0.27–0.50; *p* < 0.001) (Table 5). Annual MDR rates varied over the course of the study, according to an analysis of temporal trends in MDR (Figure 2).

**Table 4:** Association between age at onset and infection type.

Infection type	OR	95% CI	*p*-value
Gram-negative vs. Gram-positive	1.56	1.16–2.10	0.003
*Candida spp* vs. Gram-positive	3.70	1.63–8.37	0.002
Gram-positive vs. others	0.59	0.44–0.78	<0.001

Odds ratios (OR) and 95% confidence intervals (CI) were calculated using univariate logistic regression analysis.

**Table 5:** Independent predictors of multidrug resistance (multivariate logistic regression analysis).

Variable	Adjusted OR	95% CI	*p*-value
Gram-negative vs. Gram-positive	0.37	0.27–0.50	**<0** **.** **001**
≥72 h (LOS)	2.53	1.84–3.47	**<0** **.** **001**
Year (per year increase)	1.06	0.98–1.14	0.169
Male sex	1.05	0.79–1.41	0.731

Multivariate logistic regression analysis was performed to identify independent predictors. Adjusted odds ratios (OR) and 95% confidence intervals (CI) are presented. Bold values indicate statistically significant associations at *p* < 0.05 in the multivariable logistic regression analysis.

### Temporal Trends in Multidrug Resistance

Supplementary Figure S1 shows species-specific temporal trends in MDR prevalence among the most clinically significant infections. In most years, Gram-positive bacteria had higher MDR rates.

### Trends in Antibiotic Resistance Over Time

Carbapenem resistance was the only antibiotic resistance trend to exhibit a statistically significant rise over time (*p* for trend = 0.004). Colistin, piperacillin–tazobactam, ampicillin–sulbactam, ceftriaxone, gentamicin, vancomycin, teicoplanin, and clindamycin showed no discernible temporal alterations (Table 6).

**Table 6:** Temporal trends in resistance rates of selected antibiotics (%).

Antibiotic	2018	2019	2020	2021	2022	2023	2024	*P* for trend
Carbapenem	4.5	6.4	18.8	27.3	23.6	17.2	25.0	0.004
Colistin	12.5	0.0	0.0	2.8	14.3	5.9	10.0	0.600
Piperacillin-tazobactam	15.3	14.8	13.2	16.0	18.4	19.1	17.8	0.210
Ampicillin-sulbactam	30.0	28.5	27.9	32.5	35.2	38.0	36.7	0.095
Ceftriaxone	68.2	68.3	71.4	90.0	100.0	75.0	60.0	0.812
Gentamicin	60.5	40.0	0.0	NA	50.0	28.0	55.2	0.178
Vancomycin	0.0	0.9	0.0	0.0	3.2	0.0	1.7	0.202
Teicoplanin	0.0	0.0	0.0	3.1	0.0	NA	0.0	0.490
Clindamycin	39.1	20.6	18.6	25.0	37.9	44.4	41.1	0.080

*P* for trend values were calculated using logistic regression analysis with year treated as a continuous variable (NA, not available).

### Antimicrobial Susceptibility Profiles

None of the frequently used empirical antibiotics met the suggested ≥80% susceptibility threshold, according to an analysis of antimicrobial susceptibility profiles. The efficiency of existing empirical treatment techniques is limited, as indicated by the overall susceptibility rates remaining inadequate among commonly used medicines (Table 7).

**Table 7:** Overall susceptibility rates of first-line antibiotics.

Antibiotic	Susceptibility (%)	Interpretation
Penicillin	12.2	Not suitable
Ampicillin-sulbactam	38.4	Not suitable
Gentamicin	53.3	Limited
Ceftriaxone	27.5	Not suitable
Ceftazidime	45.7	Limited

Values represent overall susceptibility rates.

### Pathogen-Specific Multidrug Resistance Patterns

Pathogen-specific analysis showed that coagulase-negative staphylococci, Acinetobacter species, and Staphylococcus aureus had the highest MDR rates.In contrast, Pseudomonas species and Escherichia coli showed comparatively lower MDR rates. Overall, MDR was significantly more common among Gram-positive organisms than Gram-negative organisms, with rates of 55.9% and 35.5%, respectively (*p* < 0.001), as shown in Table 8.

**Table 8:** Pathogen-specific and group-level distribution of multidrug resistance (MDR) Among bacterial isolates, *p*-value (Gram-positive vs. Gram-negative) <0.001.

Pathogen	Total isolates (*n*)	MDR-positive (*n*)	MDR rate (%)	*p*
Gram-positive	472	264	55.9	<0.001
Gram-negative	366	130	35.2	
Overall	838	394	47	
*Coagulase-negative Staphylococcus*	281	171	60.9	
*Staphylococcus aureus*	158	91	57.6	
*Escherichia coli*	154	44	28.6	
*Klebsiella spp.*	114	59	51.8	
*Pseudomonas spp.*	45	5	11.1	
*Enterobacter spp.*	23	8	34.8	
*Enterococcus spp.*	22	0	0.0	
*Acinetobacter spp.*	18	11	61.1	
*Streptococcus spp.*	6	0	0.0	
*Serratia spp.*	5	2	40.0	
*Micrococcus spp.*	5	2	40.0	
*Citrobacter spp.*	3	1	33.3	
*Proteus spp.*	3	0	0.0	
*Stenotrophomonas spp.*	1	0	0.0	

Values are presented as a number (*n*) and a percentage (%). MDR was defined as non-susceptibility to at least one agent in three or more antimicrobial categories. Gram-positive and Gram-negative totals (*n* = 838) represent all bacterial isolates included in the analysis after exclusion of contaminants and duplicates. A *p*-value <0.05 was considered statistically significant. “<0.001” indicates not applicable for subgroup-level comparisons.

## Discussion

This six-year NICU-based study offers a thorough summary of the dynamics of antimicrobial resistance (AMR), pathogen dispersion, and neonatal sepsis epidemiology in a context with limited resources. The 30% overall culture positivity rate is consistent with earlier research on newborn sepsis in comparable neonatal intensive care unit (NICU) settings, where positivity ranges from 20% to 40%, indicating both the actual infection burden and the limitations of blood culture sensitivity in neonates (25, 26). 11–19% of all newborn deaths worldwide are caused by neonatal sepsis, which disproportionately affects low- and middle-income countries (LMICs) (27, 28). The persistent global The burden of neonatal infection is further supported by recent data from Frontiers in Pediatrics, which shows that culture-confirmed neonatal sepsis is still common in LMIC NICUs, with rates exceeding 25% in some tertiary facilities (29).

In contrast to several LMIC-based NICU studies where Gram-negative organisms predominate in the pathogenesis of newborn sepsis, this study shows a predominance of Gram-positive organisms (53.0%) (30, 31). For instance, almost 70% of isolates in a multicenter NICU research were Gram-negative pathogens, namely Klebsiella pneumoniae and E. coli, which are closely linked to infant mortality and rapid clinical deterioration (30, 32). The current study, on the other hand, found that the most common pathogen was coagulase-negative staphylococci (CoNS), followed by Staphylococcus aureus, E. coli, and Klebsiella spp. This trend is comparable with hospital-acquired NICU infections and device- related sepsis that have been documented worldwide (33).

The high incidence of CoNS, however, needs to be viewed with caution. CoNS are both prevalent infections and frequent contaminants in newborn microbiology, especially in environments with inaccurately standardized blood culture collection procedures. (34). Up to 30% of CoNS-positive cultures may indicate contamination rather than actual bacteremia, a restriction that has been extensively emphasized in newborn infection studies (34). However, their prevalence in NICUs is also becoming more widely acknowledged as a sign of the usage of invasive devices and bloodstream infections linked to central lines. (35).

Late-onset sepsis (LOS) was significantly more common and linked to higher culture positivity than EOS (37.7% vs. 20.7%, *p* < 0.001), despite the lack of a significant correlation between sex and infection timing (*p* = 0.548). This result is consistent with global NICU data showing that extended hospital stays, invasive procedures, and antibiotic exposure are the main causes of LOS. (36, 37). The observed microbial shift increased Gram-negative and fungal infections in LOS, and Gram-positive dominance in EOS is biologically reasonable and compatible with known mechanisms of newborn infection (38). LOS represents horizontal transmission from hospital settings and medical equipment, whereas EOS is mostly associated with vertical transmission from maternal colonization (39). Candida species were found (5.9%). The results of NICU research, which show that extended antibiotic exposure and central line use greatly raise the risk of fungal sepsis, are especially worrisome in LOS (40).

Antimicrobial pressure, infection control procedures, and potential outbreak clusters may have an impact on the changing NICU microbial ecology, as seen by the considerable variation in pathogen distribution over time (*p* < 0.001). Nonetheless, the lasting predominance of CoNS, Staphylococcus aureus, Klebsiella, and E. coli over time indicates stable endemic colonization within NICU settings, which is in line with studies of long-term persistence of NICU-adapted strains worldwide (41). Despite variations in resistance patterns, recent Frontiers in Pediatrics research shows stable pathogen profiles in NICUs over long monitoring periods, indicating that hospital reservoirs and biofilm-associated colonization are important factors in persistent transmission dynamics (42).

LOS was independently associated with multidrug resistance (aOR: 2.53; *p* < 0.001), confirming its role as a major driver of resistant neonatal infections. This is consistent with global NICU literature showing that prolonged hospital exposure and repeated antibiotic administration significantly increase MDR risk (43).

Importantly, no association was found between study year and MDR trends after adjustment (*p* = 0.169), suggesting that resistance pressure is chronically established rather than progressively worsening. Similar findings have been reported in NICU AMR studies where resistance patterns remain stable over time despite changing antimicrobial policies, indicating weak stewardship enforcement (44). Weak IPC systems and limited monitoring capacity across Somali health facilities likely contribute to the observed burden of neonatal infections and antimicrobial resistance, as only 6.6% of facilities report routine IPC monitoring practices (52).

The significantly higher MDR burden in Gram-positive organisms (55.9% vs. 35.5%, *p* < 0.001) differs from many LMIC studies where Gram-negative MDR predominates (45). This may reflect heavy empirical use of vancomycin and beta-lactam antibiotics, particularly in tertiary NICUs with high device utilization. CoNS and S. aureus MDR predominance has also been documented in NICU studies from Asia and Europe, particularly in central line-associated bloodstream infections (46).

The observed increases in carbapenem resistance (*p* for trend = 0.004) is highly concerning. Carbapenem-resistant Enterobacterales are recognized globally as critical priority pathogens by the WHO due to their limited treatment options and high mortality in neonates (47). Similar increasing trends have been reported in multicounty NICU surveillance studies, highlighting the global expansion of carbapenem resistance in neonatal units (48).

Critically, none of the commonly used empirical antibiotics achieved ≥80% susceptibility, indicating a major mismatch between empirical treatment guidelines and local resistance patterns. This mirrors findings from Frontiers and BMJ Global Health studies, which show a declining efficacy of the WHO-recommended first-line neonatal antibiotics (ampicillin + gentamicin) in LMIC settings (27, 49).

Similar issues, such as high MDR prevalence (up to 61%) and Klebsiella pneumoniae dominance in neonatal sepsis, are reported in recent pediatric NICU studies from developing countries. Additionally, a 2025 Frontiers study showed a substantial correlation between neonatal sepsis and longer hospital stays, higher expenses, and treatment failure due to AMR (42). Similarly, comprehensive reviews verify that the misuse of broad-spectrum antibiotics and inadequate diagnostic systems are contributing to the global rise in AMR in newborn sepsis, especially in LMICs (49). However, the current study shows a more severe resistance ecology, indicating both stronger antimicrobial pressure and worse stewardship mechanisms, in contrast to high-income nation NICUs, where CoNS is dominant but MDR rates are lower (44).

The study's extended six-year monitoring period, which enables a thorough assessment of temporal trends in pathogen distribution and resistance patterns, is its main strength. Nevertheless, there are certain drawbacks, including the inability to distinguish between genuine infections and CoNS contamination, the lack of molecular typing to verify clonal transmission, and the absence of clinical outcome information, such as death and duration of stay. Furthermore, detection and resistance reporting may have been impacted by any variations in laboratory procedures over time.

### Policy implications

The results of this six-year NICU surveillance study have significant consequences for infection prevention and control (IPC) practices, antibiotic stewardship, and neonatal health policy in Somalia and other low-resource environments. Rising rates of carbapenem resistance, multidrug resistance (MDR), and late-onset sepsis (LOS) underscore important systemic deficiencies that require immediate policy attention at the hospital and national levels.

## Conclusion

This study shows a significant prevalence of MDR newborn infections linked to LOS, together with rising carbapenem resistance and persistently low responsiveness to common empirical treatments. The necessity for antimicrobial stewardship programs, improved infection prevention and control (IPC) systems, and ongoing NICU-based microbiological surveillance to direct empirical therapy in settings with low resources is clearly supported by our findings.

## Data Availability

The original contributions presented in the study are included in the article/Supplementary Material, further inquiries can be directed to the corresponding author.
