# Supplementary material for: Microbial etiology and antimicrobial resistance patterns in neonates admitted to a tertiary neonatal intensive care unit in Mogadishu, Somalia
Source: Front Pediatr. 2026 Aug 18;14:1888678. doi: 10.3389/fped.2026.1888678 (PMC13539597; doi:10.3389/fped.2026.1888678)
Supplement: Supplementary file 1 [file Supplementaryfile1.docx]

**Supplementary Material**

**Article title:** *Microbial etiology and antimicrobial resistance patterns in neonates admitted to a tertiary neonatal intensive care unit in Mogadishu, Somalia*

**Supplementary Figure S1. Temporal trends in multidrug resistance among the six most frequently isolated bacterial species during the study period (2018–2024).**

**Supplementary Figure S1.** Annual multidrug resistance (MDR) prevalence among the six most frequently isolated bacterial species between 2018 and 2024. MDR was defined according to the international consensus criteria proposed by Magiorakos et al. Trends are presented separately for each bacterial species and should be interpreted with caution for years with limited isolate numbers.

**Supplementary Figure S2. Most frequent antimicrobial resistance patterns among multidrug-resistant isolates according to bacterial species**

**Supplementary Figure S2.** Antimicrobial susceptibility profiles of the most frequently isolated bacterial species included in the study.
